# First Insight into the Molecular Epidemiology of* Mycobacterium tuberculosis* Isolates from the Minority Enclaves of Southwestern China

**DOI:** 10.1155/2017/2505172

**Published:** 2017-05-17

**Authors:** Lianyong Chen, Yu Pang, Li Ma, Huijuan Yang, Haohao Ru, Xing Yang, Shuangqun Yan, Mao Jia, Lin Xu

**Affiliations:** ^1^Yunnan Provincial Center for Tuberculosis Research, Yunnan Provincial Center for Disease Control and Prevention, Kunming, China; ^2^Yunnan Provincial Dispensary for Tuberculosis Control and Prevention, Yunnan Provincial Center for Disease Control and Prevention, Kunming, China; ^3^National Clinical Laboratory on Tuberculosis, Beijing Key Laboratory on Drug-Resistant Tuberculosis Research, Beijing Chest Hospital, Beijing Tuberculosis and Thoracic Tumor Institute, Capital Medical University, Beijing, China

## Abstract

Yunnan is a province located in southwestern China. The aim of this study was to investigate the genetic diversity of* Mycobacterium tuberculosis* (MTB) strains circulating in Yunnan Province. We used spoligotyping and a 12-locus high-resolution VNTR set to identify a total of 271 MTB isolates collected from six prefectures' Yunnan. All the 271 patients were classified as 11 different ethnic groups, including 133 Han patients (49.1%) and 138 minority patients (50.9%). Spoligotyping analyses revealed that the largest two spoligotypes were SIT1 (Beijing family, *n* = 136) and SIT53 (T family, *n* = 35). Statistical analysis indicated that the proportion of Beijing genotype in Qujing was significantly higher than that in the ethnic enclaves (*P* ≤ 0.01). Compared with the percentage of Beijing-family isolates from patients under 25 years of age (72.7%), those from patients aged 45~64 years (44.6%, *P* < 0.01) and older than 64 years (48.1%) (*P* = 0.04) were significantly lower. Beijing genotype strains (23.8%, 36/151) showed higher clustering rate than non-Beijing genotype strains (16/120, 13.3%, *P* = 0.029). In conclusion, our data demonstrated that the Beijing genotype was the predominant genotype in Yunnan Province. The distribution of Beijing genotype strains showed geographic diversity. In addition, Beijing genotype was associated with recent transmission rather than drug resistance.

## 1. Introduction

Tuberculosis (TB), caused by* Mycobacterium tuberculosis* complex (MTBC), remains a major global health concern and ranks as the second leading cause of death from infectious diseases [1]. The World Health Organization (WHO) estimates that TB caused 9.6 million new cases and 1.5 million deaths in 2014 [2]. Only behind India and Indonesia, China has the world's third-largest amount of TB patients, with an approximately 1.0 million new cases reported annually [2]. According to the most recent national TB epidemiological survey, the prevalence of pulmonary TB was 442 cases per 100,000 population in China [3]. Despite the decreased trend in the incidence and mortality rate from 1990 to 2010, TB is still one of the most important issues threatening the public health and social development in China [3, 4].

Molecular epidemiological investigation has been proved to be a useful tool for TB control and surveillance, which allows us to better understand the dynamic of disease transmission and distinguish between relapse and infection [5]. In addition, there is increasing evidence that certain genotypes of* M. tuberculosis* are associated with drug resistance, virulence, and disease progression [6]. Hence, the knowledge regarding strain diversity within the patients has special contemplation during TB epidemics or suspected outbreaks, thereby providing new insights for establishing strategic measures for TB control and prevention [7, 8].

Yunnan is located in southwestern China, with an area of 394,100 square kilometers and a population of 46.9 million people in 2013. Out of all inhabitants, there are remarkable 14.0 million inhabitants belonging to 55 different ethnic minority groups, accounting for one-third of the whole inhabitants. In addition, most of these minority inhabitants live along the border with Myanmar, Laos, and Vietnam. According to the data from provincial surveillance system for TB, the overall incidence of pulmonary TB was 56 cases per 100,000 population in Yunnan [9]. The diversity of TB burden was also observed across the different regions, and the TB incidences in several regions of ethnic enclaves were three times higher than the average incidence [9]. Therefore, it is meaningful to investigate the patient's structure and drug susceptibility of MTB isolates circulating in Yunnan Province to highlight the geographic diversity of TB in ethnic enclaves. Unfortunately, limited data have been reported on this issue. In this study, our aim was to characterize the genotypes of MTB strains collected in Yunnan and to investigate the transmission profile of the predominant TB genotypes in this area.

## 2. Materials and Methods

### 2.1. Ethics Statement

This study was approved by the Ethics Committee of the Yunnan Provincial Center for Disease Control and Prevention. Patients were enrolled in the study after agreeing to and signing an Informed Consent form.

### 2.2. Bacterial Isolates

All smear-positive pulmonary TB patients seeking health care at six prefectural TB dispensaries in 2014 were continuously enrolled in this study, including Qujing, Lijiang, Dehong, Lincang, Puer, and Xishuangbanna. Demographic information was collected from Informed Consent forms. Two sputum samples from each patient were used for mycobacteria culturing with Löwenstein-Jensen (L-J) medium. One strain from each patient was collected for further molecular epidemiological study.

### 2.3. DNA Extraction

The crude genomic DNA of MTB isolates were extracted as previously reported [5]. Briefly, a loopful of colonies from L-J slants were transferred to a microcentrifuge tube containing 500 *μ*L Tris-EDTA (TE) buffer. Followed by heating at 95°C water bath for 30 min, the suspension was centrifuged at 12000 rpm for 10 min. The DNA in the supernatant was pipetted into a microcentrifuge tube for further PCR amplification.

### 2.4. Drug Susceptibility Testing (DST)

The proportional method was performed to determine the in vitro drug susceptibility of MTB isolates against isoniazid (INH) and rifampicin (RFP) according to the recommendations by the World Health Organization [4, 9]. The concentrations of INH and RFP in the L-J medium were 0.2 *μ*g/mL and 40 *μ*g/mL, respectively. The resistance of a strain against a drug was declared when the growth rate was higher than 1% compared with the control. The Yunnan Provincial TB Reference Laboratory has passed the DST proficiency tests of the National TB Reference Laboratory since 2007. Multiple drug resistant tuberculosis (MDR-TB) isolates were defined as strains being resistant to both INH and RFP.

### 2.5. Genotyping

Spoligotyping was performed using commercial kit manufactured by Isogen Bioscience BV (Maarssen, Netherlands) [4]. The genomic DNA was amplified with primers DRa (5′-CCGAGAGGGGACGGAAAC-3′) and DRb (5′-GGTTTTGGGTCTGACGAC-3′). The amplicons were hybridized with a membrane and the final image was detected with a chemiluminescence system (Amersham, Buckinghamshire, United Kingdom). The original binary data were entered in the SITVITWEB database to obtain the Spoligotyping International Type (SIT) patterns and the sublineages of MTB isolates [10]. In addition, all the isolates were genotyped with the 12-locus VNTR set with high discriminatory power described by Luo et al. [11]. The PCR products were analyzed using 1.5% agarose electrophoresis at 5 V/cm for 1 hour. The 100 bp DNA ladder and amplicons of H37Rv were loaded per 7 lanes as a size marker to calculate the repeat number of each locus. The Hunter-Gaston Discriminatory Index (HGDI) was used to analyze the discriminatory power of each VNTR locus [12]. In addition, the genotyping patterns were clustered with BioNumerics software version 5.0 (Applied Maths, Sint-Martens-Latem, Belgium).

### 2.6. Data Analysis

A chi-square test was performed to detect any significant difference between the two groups, and the statistical results were expressed as odds ratios (ORs) with 95% confidence intervals (CIs). Two-sided *P* value of <0.05 was declared as statistically significant. All calculations were analyzed in SPSS 14.0 (SPSS Inc., USA).

## 3. Results

### 3.1. Drug Susceptibility Profiles and Demographic Characteristics of Han and Minority Patients

**Figure 1:**
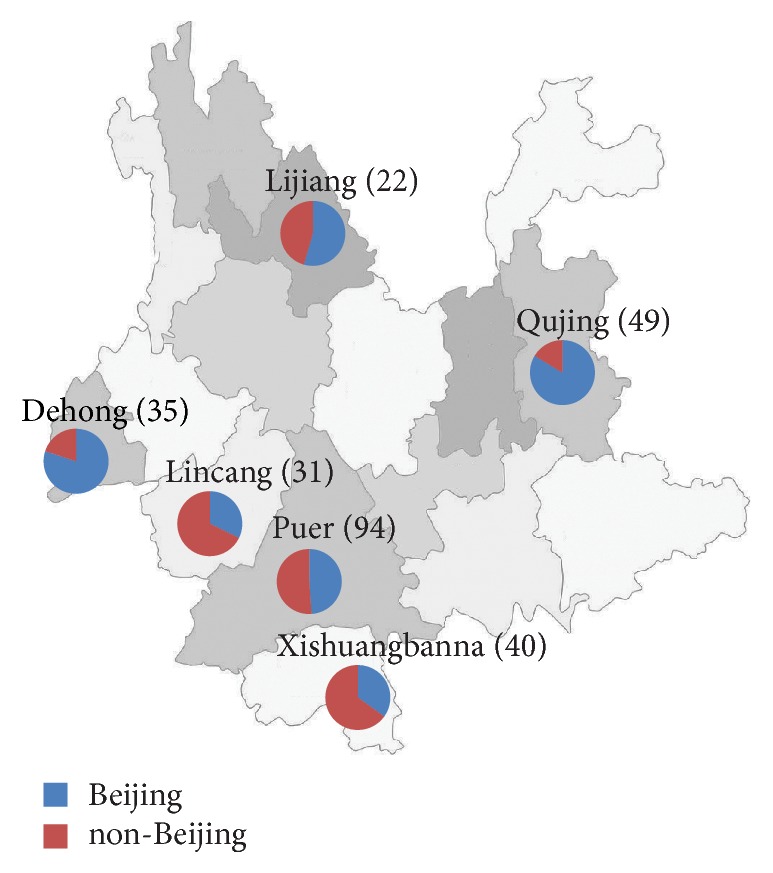
Distribution of Beijing genotype MTB isolates enrolled in this study. The number presents the absolute number of isolates in this region.

A total of 271 MTB isolates represented patients from different districts across Yunnan Province (Figure 1). Out of these patients, 196 (72.3%) were males, and 75 were females. The mean age of the patients was  49.8 ± 12.1  years (range: 16–76 years). In addition, all the 271 patients were classified as 11 different ethnic groups, including 133 Han patients (49.1%) and 138 minority patients (50.9%). Of these minority groups, Hani, Yi, and Dai were the most frequently observed, accounting for 11.1% (30/271), 10.3% (28/271), and 9.6% (26/271) of all the patients, respectively. We further analyzed the distribution of demographic and drug susceptibility characteristics between Han and minority patients. As shown in Table 1, statistical analysis indicated that the proportion of minority patients in Qujing (2.0%, 1/49) were significantly lower than that in Puer (71.3%, 67/94), Xishuangbanna (70.0%, 28/40), Lijiang (63.6%, 14/22), Dehong (62.8%, 2/35), and Lincang (19.4%, 6/31), respectively (*P* < 0.05). Compared with Han patients, the minority patients had higher proportion of farmers (88.4% versus 78.2%,  *P* = 0.02). In addition, we found that the Han patients were significantly associated with RIF resistance (*P* = 0.03), while the rate of INH resistance showed no statistically significant difference. Although all the MDR-TB patients belonged to Han patients, the difference in the prevalence of MDR-TB between Han and minority patients was not significant (Table 3), which was more likely to be attributed to small sample size. Demographic characteristics, including gender, age, and treatment history, showed no statistical difference between Han and minority patients.

### 3.2. Spoligotyping

The 271 MTB isolates were analyzed by spoligotyping in this study. The 271 genotyped strains were divided into 16 types shared by 2 to 136 isolates and 24 orphans (genotypes found only once in study). Out of these spoligotypes, SIT1 was the largest lineage (50.2%, 136/271), which belonged to the classical Beijing genotype. In addition, the second largest type was SIT53 from T1 family that included 35 isolates (12.9%, Table 2). Eleven types, containing 21 isolates, were identified for the first time.

Based on spoligotyping, 151 (55.7%) MTB strains belonged to the Beijing genotype, while the other 120 (44.3%) were from non-Beijing families, indicating that Beijing genotype was the predominant sublineage in Yunnan. Strains assigned to non-Beijing family included 45 from T1 family (16.6%), 34 from T3 family (12.5%), 6 from LAM9 family (2.2%), 5 from the T2 family (1.8%), 4 from T2-T3 family (1.5%), 2 from MANU2 family (0.7%), 1 from T5 family (0.4%), 1 from H3 family (0.4%), 1 from X2 family (0.4%), and 21 of undefined genotypes (7.7%, Table 2).

### 3.3. Drug Susceptibility Profiles and Demographic Characteristics of Beijing and Non-Beijing MTB Isolates

We compared the proportion of drug susceptibility profiles and demographic characteristics of patients identified as infection with Beijing and non-Beijing genotypes. Statistical analysis indicated that the distribution of Beijing genotype showed geographic diversity, and the proportion of Beijing genotype in Qujing was significantly higher than that in the ethnic enclaves (*P* ≤ 0.01). Compared with the percentage of Beijing-family isolates from patients under age of 25 years, those from patients aged 45~64 years [OR (95% CI): 0.30 (0.14~0.66), *P* < 0.01] and older than 64 years [OR (95% CI): 0.35 (0.13~0.95), *P* = 0.04] were significantly lower. With regard to the gender distribution of Beijing genotype strains, the percentage of female patients infected with Beijing genotype strains was significantly higher than that of male patients, indicating that women might be at high risk for the infection of Beijing genotype strains [OR (95% CI): 2.04 (1.17~3.57), *P* = 0.01]. Our data revealed that occupation, treatment history, and drug resistance showed no statistically significant association with the Beijing genotype (Figure 1 and Table 3).

### 3.4. VNTR

**Figure 2:**
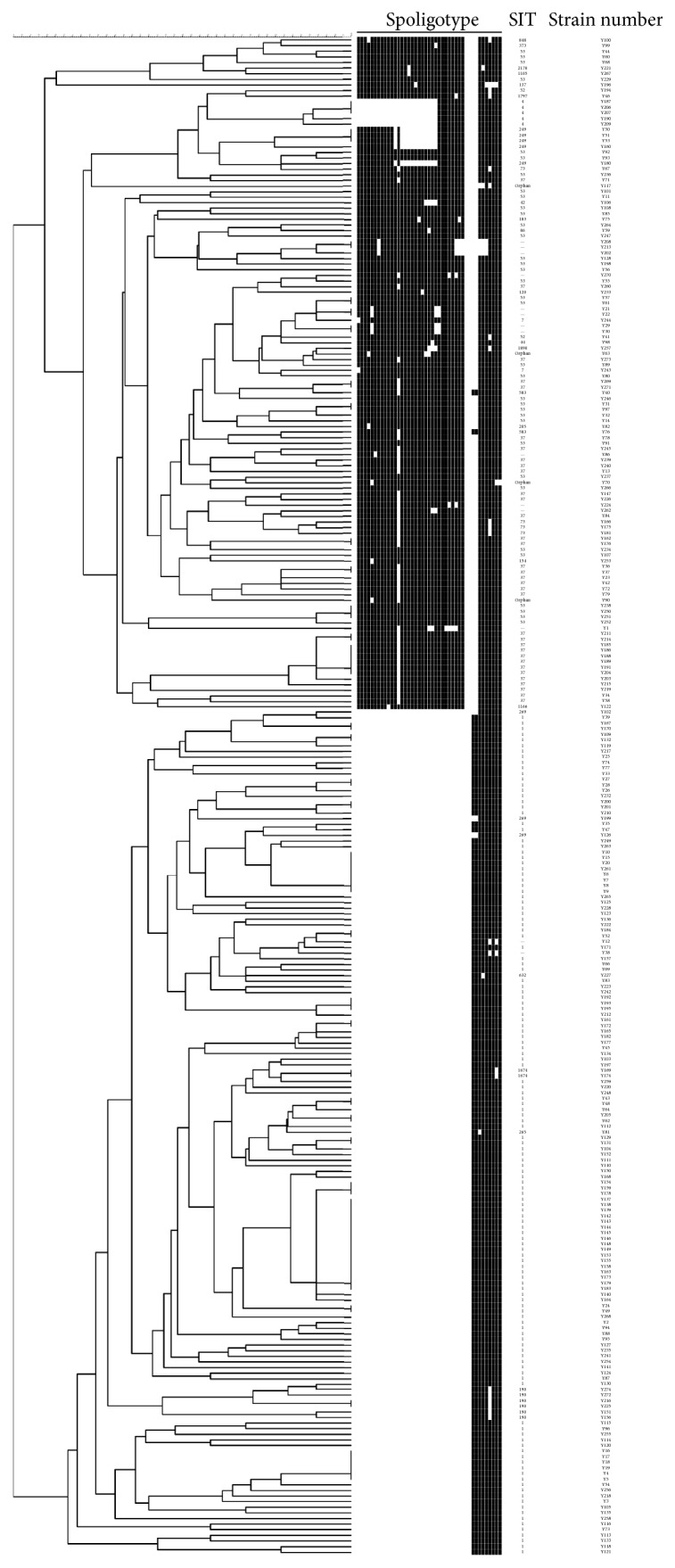
Dendrogram of 271 MTB isolates from Yunnan Province. The phylogenetic tree was generated with the 12-VNTR loci set.

Using the 12-locus high-resolution VNTR method, the 271 isolates were differentiated into 30 clusters (2 to 17 isolates per cluster) and 177 unique genotypes, showing a clustering rate of 23.6% (Figure 2 and Table  S1 in Supplementary Material available online at https://doi.org/10.1155/2017/2505172). We also analyzed the clustering rate according to different patients. Our data revealed that the clustering rate of Han patients (26.3%, 35/133) was significantly higher than that of minority patients (15.2%, 21/138,  *P* = 0.024). In addition, Beijing genotype strains (23.8%, 36/151) showed higher clustering rate than non-Beijing genotype strains (16/120, 13.3%,  *P* = 0.029). Interestingly, the largest cluster with 6 3 8 2 4 8 5 3 3 8 4 2 VNTR profile contained 17 strains, which were all from Han patients in Qujing district. As shown in Table 4, eight out of the 12 VNTR loci, including Qub11b, Qub18, Qub26, MIRU26, Mtub21, Mtub04, ETR-F, and MIRU31, were highly discriminating loci, the allelic diversity for which was higher than 0.6 [13]. All the other 4 loci (MIRU10, Qub4156, MIRU40, and Qub1895) showed moderate discrimination (0.3 ≤ HGDI ≤ 0.6). In addition, we found that the loci exhibited different discriminating ability to distinguish Beijing and non-Beijing genotype strains. Qub26, Mtub04, and MIRU40 showed higher discriminating power for non-Beijing genotype strains, while Mtub21 and Qub4156 were more effective in identifying Beijing genotype strains (Table 4).

## 4. Discussion

This study provides the first insight into the molecular epidemiological characteristics of MTB strains circulating in Yunnan Province, China. Our data revealed that Beijing genotype is still the predominant genotype throughout Yunnan, accounting for 55.7% of the MTB strains in the present study. The prevalence of Beijing genotype is similar to that in Fujian (57.3%) [14] and that in Guangxi (61.9%) [15], although it is lower than most regions in China, ranging from 76.7% to 91.9% [16–19]. All these findings were consistent with a recent study by Pang et al. (2012) [4], which revealed that the distribution of Beijing differed from one geographic region to another in China, and the Beijing genotype constitutes 76.5% of the MTB strains currently epidemic in north China, with only 53.2% of those in south China. Although the exact reason for the relative low prevalence of Beijing genotypes in south China remains elusive, it is believed to be associated with the natural environment, living habit, and the evolution history of Beijing genotype [4, 20].

In addition, we also found that the distribution of Beijing genotype showed remarkable geographic diversity in Yunnan, which may be attributed to the different composition of minority patients among these regions. The prevalence of Beijing genotype in the Han enclaves was significantly higher than that in the minority enclaves, which was consistent with the observation from Luo and colleagues [20]. In Yunnan, as opposed to Han patients, the minority patients generally live in the mountainous areas located in the national boundary lines rather than the plain areas. Hence, the lower patient densities of mountainous areas may play an important role in decreasing the transmission of the epidemic Beijing lineage. In addition, due to living in the regions bordering Vietnam and Myanmar, the minority patients prefer to move across national boundary lines for commercial trade. Considering the frequent patient communication between minority patients and neighboring foreigner, the relatively low prevalence of Beijing genotype in Vietnam (35%) [21] and Myanmar (32%) [22] may be another potential reason responsible for our findings.

Several previous studies demonstrated that the Beijing genotype is associated with young patients (aged less than 25 years) [21, 23], while researchers from other countries observed no association between the infection with the Beijing genotype and a patient age [24]. In line with the former results, we also found that young age was a risk factor for the infection with Beijing genotype strain. On one hand, compared with the older patients, children and young adults undergo higher level of social activities [25]. In view of the increased transmissibility of Beijing genotype [26], the more frequent social interactions may cause the recent transmission of Beijing strains in these patients. On the other hand, BCG vaccination was included in the National Immunization Program of China since 1980s [27]. Previous studies have hypothesized that Beijing strains could escape from insufficient protection induced by BCG [28, 29], which may be responsible for their emergence in the BCG-vaccinated young patients. In addition to age, we observed that infection with Beijing genotype was associated with gender, while the exact reason for this difference still remains unknown.

A recent report from Yang and colleagues (2015) has demonstrated that Beijing genotype strains favor transmission in China [26], which is consistent with our observation that the rate of Beijing genotype was significantly higher than that of non-Beijing genotype. Considering the lower prevalence of Beijing genotype strains in the minority patients, it also serves as the dominant contributor to the lower clustering rate of TB strains in these patients. Interestingly, we also identified a suspected outbreak in Qujing of Yunnan Province, which included 17 Beijing genotype strains sharing the same VNTR pattern. Further epidemiological investigation will help us to identify the “superspreader” of this outbreak. Recently, numerous studies have declared that VNTR can not capture the full level of genetic diversity within a single MTB genotype, while Whole-Genome Sequencing (WGS) serves as a useful tool to identify MTB isolates associated with tuberculosis outbreak due to its inherent advantages exceeding conventional molecular epidemiologic techniques [30, 31]. Nevertheless, our genotyping data using VNTR reflect the potential ongoing community transmission in Yunnan, which highlights the urgent need for early diagnosis of the infectious TB cases to avoid a large TB outbreak. In recent years, more than 70% of TB laboratories in Yunnan have been equipped with the molecular diagnosis tools, including GeneXpert and LAMP, and further the application of molecular techniques with high sensitivity for TB diagnosis will increase access to testing and decrease diagnostic delays in a resource-limited county, thereby decreasing the transmission of TB in the community.

In conclusion, our data demonstrated that the Beijing genotype was the predominant MTB genotype in Yunnan Province. The prevalence of Beijing genotype strains in the Han enclaves was significantly higher than that in the minority enclaves, indicating that distribution of TB strain showed geographic diversity. In addition, we observed that Beijing genotype was more likely to contribute to recent transmission rather than drug resistance. The suspected TB outbreak identified by VNTR highlights the urgent need for early diagnosis of the infectious TB cases to avoid a large TB outbreak.

## Supplementary Material

Supplementary Table 1 shows demographic characteristics, spoligotypes, and VNTR profiles of MTB isolates enrolled in this study.

## Figures and Tables

**Table 1:** Differences of characteristics between Han and minority population.

Characteristics	Total number of isolates	Number (%) of isolates		OR	95% CI	*P* value
		Han	Minority			
		(*n* = 133)	(*n* = 138)			
Resistance to						
INH	14 (5.2)	9 (6.8)	5 (3.6)	0.52	0.17~1.59	0.24
RIF	5 (1.8)	5 (3.8)	0 (0.0)	0.96	0.93~0.99	0.03
MDR	4 (1.5)	4 (3.0)	0 (0.0)	0.97	0.94~1.00	0.06
Gender						
Men	196 (72.3)	94 (70.7)	102 (73.9)	1.0 (Ref.)	—	—
Women	75 (27.7)	39 (29.3)	36 (26.1)	1.18	0.69~2.00	0.55
Age group (years)						
<25	44 (16.2)	21 (15.8)	23 (16.7)	1.0 (Ref.)	—	—
25–44	108 (39.9)	54 (40.6)	54 (39.1)	1.10	0.54~2.21	0.80
45–64	92 (33.9)	45 (33.8)	47 (34.1)	1.05	0.51~2.15	0.90
>64	27 (10.0)	13 (9.8)	14 (10.1)	1.02	0.39~2.65	0.97
Occupation						
Farmer	226 (83.4)	104 (78.2)	122 (88.4)	0.47	0.24~0.91	0.02
Others	45 (16.6)	29 (21.8)	16 (11.6)	1.0 (Ref.)	—	—
Region						
Qujing	49 (18.1)	48 (36.1)	1 (0.7)	1.0 (Ref.)	—	—
Dehong	35 (12.9)	13 (9.8)	22 (15.9)	0.01	0.00~0.10	<0.01
Lijiang	22 (8.1)	8 (6.0)	14 (10.1)	0.01	0.00~0.10	<0.01
Lincang	31 (11.4)	25 (18.8)	6 (4.3)	0.09	0.10~0.76	0.01
Puer	94 (34.7)	27 (20.3)	67 (48.6)	0.01	0.00~0.07	<0.01
Xishuangbanna	40 (14.8)	12 (9.0)	28 (20.3)	0.01	0.00~0.07	<0.01
Treatment History						
New case	246 (90.8)	120 (90.2)	126 (91.3)	1.0 (Ref.)	—	—
Retreated	25 (9.2)	13 (9.8)	12 (8.7)	1.14	0.50~2.59	0.76

**Table 2:** Spoligotypes of the 271 *M. tuberculosis* isolates enrolled in this study.

SIT^a^	Lineage^b^	Number (%)	Spoligotype description binary
1	Beijing	136 (50.2)	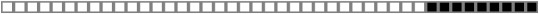
190	Beijing	6 (2.2)	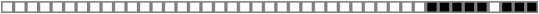
269	Beijing	3 (1.1)	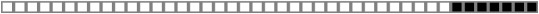
1674	Beijing	2 (0.7)	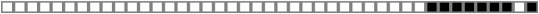
632	Beijing	1 (0.4)	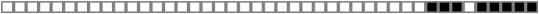
265	Beijing	1 (0.4)	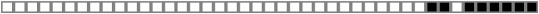
743	Beijing	2 (0.7)	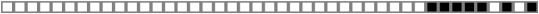
53	T1	35 (12.9)	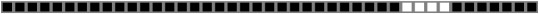
7	T1	2 (0.7)	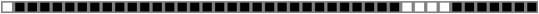
86	T1	1 (0.4)	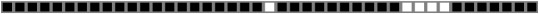
120	T1	1 (0.4)	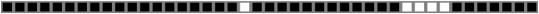
154	T1	1 (0.4)	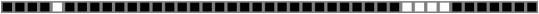
205	T1	1 (0.4)	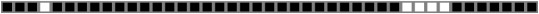
373	T1	1 (0.4)	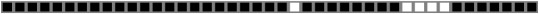
1105	T1	1 (0.4)	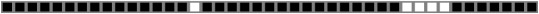
1166	T1	1 (0.4)	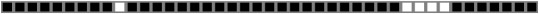
2170	T1	1 (0.4)	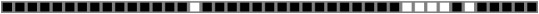
52	T2	2 (0.7)	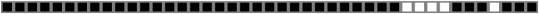
848	T2	1 (0.4)	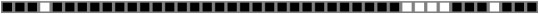
1797	T2	1 (0.4)	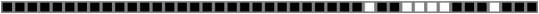
1890	T2	1 (0.4)	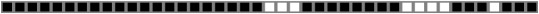
73	T2-T3	4 (1.5)	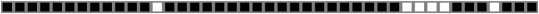
37	T3	34 (12.5)	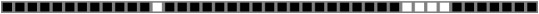
44	T5	1 (0.4)	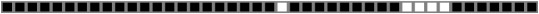
183	H3	1 (0.4)	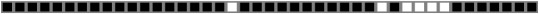
42	LAM9	1 (0.4)	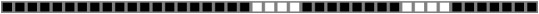
249	LAM9	5 (1.8)	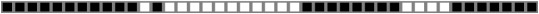
583	MANU2	2 (0.7)	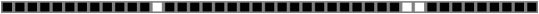
137	X2	1 (0.4)	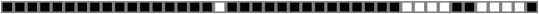
Orphan	NA	5 (1.8)	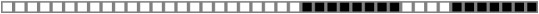
Orphan	NA	4 (1.5)	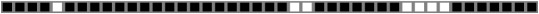
Orphan	NA	3 (1.1)	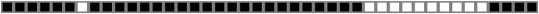
Orphan	NA	2 (0.7)	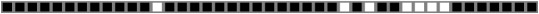
Orphan	NA	1 (0.4)	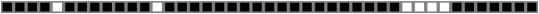
Orphan	NA	1 (0.4)	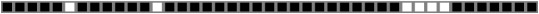
Orphan	NA	1 (0.4)	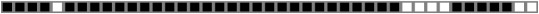
Orphan	NA	1 (0.4)	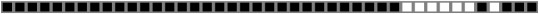
Orphan	NA	1 (0.4)	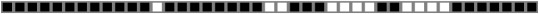
Orphan	NA	1 (0.4)	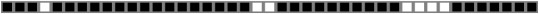
Orphan	NA	1 (0.4)	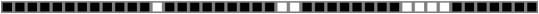

^a^SIT from SITVITWEB database.

^b^Representing spoligotype lineages annotated in SITVITWEB database.

Number of strains with the same SIT.

Orphan represents the spoligotyping type which is not found in SITVITWEB database.

**Table 3:** Differences of characteristics between Beijing and non-Beijing genotype.

Characteristics	Total number of isolates	Number (%) of isolates		OR	95% CI	*P* value
		Beijing	Non-Beijing			
		(*n* = 151)	(*n* = 120)			
Resistance to						
INH	14 (5.2)	8 (5.3)	6 (5.0)	0.94	0.32~2.79	0.91
RIF	5 (1.8)	4 (2.6)	1 (0.8)	0.31	0.03~2.80	0.39
MDR	4 (1.5)	3 (2.0)	1 (0.8)	0.41	0.04~4.04	0.63
Gender						
Men	196 (72.3)	100 (66.2)	96 (80.0)	1.00 (Ref.)	—	—
Women	75 (27.7)	51 (33.8)	24 (20.0)	2.04	1.17~3.57	0.01
Age group (years)						
<25	44 (16.2)	32 (21.2)	12 (10.0)	1.00 (Ref.)	—	—
25–44	108 (39.9)	65 (43.0)	43 (35.8)	0.57	0.26~1.22	0.14
45–64	92 (33.9)	41 (27.2)	51 (42.5)	0.30	0.14~0.66	<0.01
>64	27 (10.0)	13 (8.6)	14 (11.7)	0.35	0.13~0.95	0.04
Occupation						
Farmer	226 (83.4)	121 (80.1)	105 (87.5)	0.58	0.29~1.13	0.11
Others	45 (16.6)	30 (19.9)	15 (12.5)	1.00 (Ref.)	—	—
Ethnicity						
Han	133 (49.1)	76 (50.3)	57 (47.5)	1.00 (Ref.)	—	—
Hani	30 (11.1)	11 (7.3)	19 (15.8)	0.43	0.19~0.98	0.04
Yi	28 (10.3)	10 (6.6)	18 (15.0)	0.42	0.18~0.97	0.04
Dai	26 (9.6)	13 (8.6)	13 (10.8)	0.75	0.32~1.74	0.50
Others	54 (19.9)	41 (27.2)	13 (10.8)	2.37	1.16~4.82	0.02
Region						
Qujing	49 (18.1)	41 (27.2)	8 (6.7)	1.00 (Ref.)	—	—
Dehong	35 (12.9)	28 (18.5)	7 (5.8)	0.78	0.25~2.40	0.67
Lijiang	22 (8.1)	12 (7.9)	10 (8.3)	0.23	0.08~0.72	0.01
Lincang	31 (11.4)	10 (6.6)	21 (17.5)	0.09	0.03~0.27	<0.01
Puer	94 (34.7)	46 (30.5)	48 (40.0)	0.19	0.08~0.44	<0.01
Xishuangbanna	40 (14.8)	14 (9.3)	26 (21.7)	0.11	0.04~0.28	<0.01
Treatment History						
New case	246 (90.8)	138 (91.4)	108 (90.0)	1.00 (Ref.)	—	—
Retreated	25 (9.2)	13 (8.6)	12 (10.0)	0.85	0.37~1.93	0.69

**Table 4:** Allelic diversity of 12 different VNTR loci among *Mycobacterium tuberculosis* strains (*n* = 271).

Locus	HGDI		
	All strains	Beijing	Non-Beijing
Qub11b	0.830	0.731	0.714
Qub18	0.854	0.813	0.672
Qub26	0.800	0.632	0.907
MIRU26	0.799	0.655	0.832
Mtub21	0.790	0.705	0.469
Mtub04	0.647	0.397	0.604
ETR-F	0.633	0.451	0.507
MIRU31	0.630	0.277	0.334
MIRU10	0.570	0.162	0.274
Qub4156	0.533	0.625	0.228
MIRU40	0.476	0.243	0.639
Qub1895	0.363	0.368	0.356
