# Supplementary material for: First Insight into the Molecular Epidemiology of Mycobacterium tuberculosis Isolates from the Minority Enclaves of Southwestern China
Source: Biomed Res Int. 2017 May 17;2017:2505172. doi: 10.1155/2017/2505172 (PMC5449728; doi:10.1155/2017/2505172)
Supplement: Supplementary file 1 — Supplementary Table 1 shows demographic characteristics, spoligotypes, and VNTR profiles of MTB isolates enrolled in this study. [file 2505172.f1.pdf]

ID	Gender
Y1	Male
Y8	Male
Y32	Male
Y36	Male
Y39	Male
Y40	Female
Y44	Male
Y45	Male
Y49	Male
Y50	Male
Y51	Male
Y52	Male
Y53	Male
Y54	Female
Y55	Male
Y58	Male
Y67	Male
Y71	Male
Y74	Female
Y81	Male
Y83	Male
Y84	Male
Y86	Male
Y87	Male
Y89	Female
Y90	Male
Y95	Female
Y103	Male
Y104	Male
Y106	Female
Y107	Male
Y108	Male
Y111	Male
Y114	Male
Y115	Male
Y122	Male
Y123	Male
Y129	Female
Y130	Male
Y135	Female
Y136	Female
Y137	Male
Y138	Female
Y139	Female
Y140	Female
Y141	Female

Y142	Female
Y143	Male
Y144	Female
Y145	Male
Y146	Male
Y147	Male
Y148	Female
Y149	Male
Y150	Male
Y151	Male
Y152	Male
Y153	Female
Y154	Male
Y155	Male
Y156	Male
Y157	Female
Y158	Male
Y159	Male
Y160	Female
Y161	Male
Y162	Female
Y163	Male
Y164	Female
Y165	Female
Y167	Male
Y168	Male
Y169	Female
Y170	Male
Y171	Female
Y172	Male
Y173	Female
Y174	Male
Y175	Male
Y176	Female
Y177	Male
Y178	Female
Y179	Female
Y180	Male
Y181	Male
Y182	Female
Y183	Female
Y184	Male
Y185	Male
Y186	Male
Y188	Female
Y189	Female
Y190	Male

Y191	Male
Y192	Male
Y195	Male
Y196	Male
Y197	Male
Y198	Male
Y199	Male
Y200	Male
Y202	Male
Y203	Male
Y204	Male
Y205	Male
Y206	Male
Y208	Male
Y209	Male
Y211	Female
Y212	Male
Y213	Male
Y214	Male
Y215	Male
Y219	Female
Y221	Male
Y222	Male
Y223	Male
Y226	Female
Y229	Male
Y234	Male
Y240	Male
Y241	Male
Y242	Female
Y243	Male
Y246	Male
Y249	Male
Y253	Female
Y254	Male
Y255	Female
Y258	Female
Y264	Male
Y267	Male
Y269	Male

Age	Ethnicity
32	Han
31	Han
25	Han
47	Han
46	Han
62	Han
76	Han
55	Han
31	Han
50	Han
52	Han
42	Han
37	Han
18	Han
57	Han
27	Han
43	Han
51	Han
42	Han
46	Han
37	Han
40	Han
46	Han
49	Han
72	Han
82	Han
43	Han
33	Han
31	Han
22	Han
50	Han
43	Han
33	Han
18	Han
40	Han
50	Han
34	Han
28	Han
42	Han
39	Han
36	Han
30	Han
16	Han
39	Han
40	Han
36	Han

29	Han
67	Han
22	Han
20	Han
55	Han
39	Han
18	Han
60	Han
40	Han
66	Han
20	Han
24	Han
42	Han
68	Han
48	Han
36	Han
49	Han
75	Han
69	Han
17	Han
42	Han
22	Han
71	Han
22	Han
15	Han
35	Han
75	Han
52	Han
29	Han
46	Han
57	Han
33	Han
35	Han
18	Han
24	Han
33	Han
32	Han
23	Han
20	Han
45	Han
61	Han
47	Han
51	Han
41	Han
49	Han
41	Han
48	Han

47	Han
30	Han
17	Han
43	Han
57	Han
31	Han
23	Han
38	Han
55	Han
51	Han
42	Han
31	Han
28	Han
55	Han
48	Han
49	Han
48	Han
46	Han
51	Han
28	Han
61	Han
40	Han
50	Han
66	Han
24	Han
45	Han
48	Han
65	Han
52	Han
22	Han
30	Han
32	Han
51	Han
40	Han
59	Han
61	Han
27	Han
28	Han
68	Han
47	Han

Location	Classification
Puer	New case
Puer	New case
Puer	New case
Puer	New case
Puer	New case
Puer	New case
Puer	New case
Puer	New case
Puer	Retreated
Puer	New case
Puer	New case
Puer	New case
Puer	New case
Puer	New case
Puer	New case
Xishuangbanna	New case
Xishuangbanna	New case
Xishuangbanna	New case
Xishuangbanna	New case
Xishuangbanna	New case
Xishuangbanna	New case
Xishuangbanna	New case
Xishuangbanna	New case
Xishuangbanna	New case
Xishuangbanna	New case
Xishuangbanna	New case
Xishuangbanna	New case
Dehong	Retreated
Dehong	New case
Dehong	New case
Dehong	Retreated
Dehong	New case
Dehong	New case
Dehong	New case
Dehong	New case
Dehong	New case
Dehong	New case
Dehong	New case
Dehong	New case
Dehong	New case
Qujing	New case
Qujing	New case
Qujing	New case
Qujing	New case
Qujing	New case
Qujing	New case

Qujing	New case
Qujing	New case
Qujing	New case
Qujing	New case
Qujing	New case
Qujing	New case
Qujing	New case
Qujing	New case
Qujing	New case
Qujing	New case
Qujing	New case
Qujing	New case
Qujing	Retreated
Qujing	New case
Qujing	New case
Qujing	New case
Qujing	New case
Qujing	Retreated
Qujing	New case
Qujing	New case
Qujing	New case
Qujing	New case
Qujing	New case
Qujing	New case
Qujing	New case
Qujing	New case
Qujing	New case
Qujing	Retreated
Qujing	New case
Qujing	New case
Qujing	New case
Qujing	New case
Qujing	New case
Qujing	New case
Qujing	New case
Qujing	New case
Qujing	New case
Qujing	New case
Qujing	New case
Qujing	New case
Qujing	New case
Qujing	New case
Lincang	New case
Lincang	New case
Lincang	Retreated
Lincang	New case
Lincang	New case

Lincang	Retreated
Lincang	New case
Lincang	New case
Lincang	New case
Lincang	New case
Lincang	New case
Lincang	New case
Lincang	New case
Lincang	New case
Lincang	New case
Lincang	New case
Lincang	New case
Lincang	New case
Lincang	Retreated
Lincang	New case
Lincang	Retreated
Lincang	New case
Lincang	New case
Lincang	New case
Lincang	New case
Lijiang	New case
Lijiang	Retreated
Lijiang	New case
Lijiang	New case
Lijiang	New case
Lijiang	New case
Puer	Retreated
Puer	New case
Puer	New case
Puer	Retreated
Puer	New case
Puer	New case
Puer	New case
Puer	New case
Puer	New case
Puer	New case
Puer	New case
Puer	New case
Lijiang	New case
Lijiang	New case

RFP	INH
S	S
S	S
S	S
S	S
S	S
S	S
S	S
S	S
S	S
S	S
S	S
S	S
S	S
S	S
S	S
S	S
S	S
S	S
S	S
S	S
S	S
S	S
S	S
S	S
S	S
S	S
S	S
S	S
S	S
S	S
R	R
S	S
S	S
R	R
S	S
S	S
S	S
S	S
R	S
S	S
S	S
S	S
S	S
S	S
S	S
S	S

S	S
S	S
S	S
S	S
S	S
S	S
S	S
S	S
S	S
S	S
S	S
S	S
S	S
S	S
S	S
S	S
S	S
S	S
S	S
S	S
S	S
S	S
S	S
S	S
R	R
S	S
S	R
R	R
S	S
S	S
S	S
S	R
S	R
S	S
S	S
S	S
S	S
S	S
S	R
S	S
S	S
S	S
S	S
S	S
S	S
S	S
S	S

S	S
S	S
S	S
S	S
S	S
S	S
S	R
S	S
S	S
S	S
S	S
S	S
S	S
S	S
S	S
S	S
S	S
S	S
S	S
S	S
S	S
S	S
S	S
S	S
S	S
S	S
S	S
S	S
S	S
S	S
S	S
S	S
S	S
S	S
S	S
S	S
S	S
S	S
S	S
S	S

Spoligo 1	Spoligo 2
1	1
0	0
1	1
1	1
0	0
1	1
1	1
0	0
0	0
1	1
1	1
0	0
1	1
0	0
1	1
1	1
1	1
1	1
0	0
0	0
0	0
1	1
1	1
0	0
1	1
1	1
0	0
0	0
0	0
1	1
1	1
1	1
0	0
0	0
0	0
1	1
0	0
0	0
0	0
0	0
0	0
0	0
0	0
0	0
0	0
0	0

0	0
0	0
0	0
0	0
0	0
1	1
0	0
0	0
0	0
0	0
0	0
0	0
0	0
0	0
0	0
0	0
0	0
0	0
1	1
0	0
1	1
0	0
0	0
0	0
0	0
0	0
0	0
0	0
0	0
0	0
0	0
0	0
1	1
1	1
0	0
0	0
0	0
1	1
1	1
0	0
0	0
0	0
1	1
1	1
1	1
1	1
0	0

1	1
0	0
0	0
1	1
0	0
1	1
0	0
0	0
1	1
1	1
1	1
0	0
0	0
1	1
0	0
1	1
0	0
1	1
1	1
1	1
1	1
1	1
0	0
0	0
1	1
1	1
1	1
1	1
0	0
0	0
0	1
1	1
0	0
1	1
0	0
0	0
0	0
1	1
1	1
1	1

Spoligo 3	Spoligo 4
1	1
0	0
1	1
1	1
0	0
1	1
1	1
0	0
0	0
1	1
1	1
0	0
1	1
0	0
1	1
1	1
1	1
1	1
0	0
0	0
0	0
1	1
1	1
0	0
1	1
1	1
0	0
0	0
0	0
1	1
1	1
1	1
0	0
0	0
0	0
1	1
0	0
0	0
0	0
0	0
0	0
0	0
0	0
0	0
0	0
0	0

0	0
0	0
0	0
0	0
0	0
1	1
0	0
0	0
0	0
0	0
0	0
0	0
0	0
0	0
0	0
0	0
0	0
0	0
1	1
0	0
1	1
0	0
0	0
0	0
0	0
0	0
0	0
0	0
0	0
0	0
0	0
0	0
1	1
1	1
0	0
0	0
0	0
1	1
1	1
0	0
0	0
0	0
1	1
1	1
1	1
1	1
0	0

1	1
0	0
0	0
1	1
0	0
1	1
0	0
0	0
1	1
1	1
1	1
0	0
0	0
1	1
0	0
1	1
0	0
1	1
1	1
1	1
1	1
1	1
0	0
0	0
1	1
1	1
1	1
1	1
0	0
0	0
1	1
1	1
0	0
1	1
0	0
0	0
0	0
1	1
1	1
1	1

Spoligo 5	Spoligo 6
1	1
0	0
1	1
1	1
0	0
1	1
1	1
0	0
0	0
1	1
1	1
0	0
1	1
0	0
1	1
1	1
1	1
1	1
0	0
0	0
0	0
1	1
1	0
0	0
1	1
0	1
0	0
0	0
0	0
1	1
1	1
1	1
0	0
0	0
0	0
1	1
0	0
0	0
0	0
0	0
0	0
0	0
0	0
0	0
0	0
0	0

0	0
0	0
0	0
0	0
0	0
1	1
0	0
0	0
0	0
0	0
0	0
0	0
0	0
0	0
0	0
0	0
0	0
0	0
1	1
0	0
1	1
0	0
0	0
0	0
0	0
0	0
0	0
0	0
0	0
0	0
0	0
0	0
0	0
1	1
1	1
0	0
0	0
0	0
1	1
1	1
0	0
0	0
0	0
1	1
1	1
1	1
1	1
0	0

1	1
0	0
0	0
1	1
0	0
1	1
0	0
0	0
1	1
1	1
1	1
0	0
0	0
1	1
0	0
1	1
0	0
1	1
1	1
1	1
1	1
1	1
0	0
0	0
1	1
1	1
1	1
1	1
0	0
0	0
1	1
1	1
0	0
0	1
0	0
0	0
0	0
1	1
1	1
1	1

Spoligo 7	Spoligo 8
1	1
0	0
1	1
1	1
0	0
1	1
1	1
0	0
0	0
1	1
1	1
0	0
1	1
0	0
1	1
1	1
1	1
1	1
0	0
0	0
0	0
1	1
1	1
0	0
1	1
1	1
0	0
0	0
0	0
1	1
1	1
1	1
0	0
0	0
0	0
1	1
0	0
0	0
0	0
0	0
0	0
0	0
0	0
0	0
0	0
0	0

0	0
0	0
0	0
0	0
0	0
1	1
0	0
0	0
0	0
0	0
0	0
0	0
0	0
0	0
0	0
0	0
0	0
0	0
1	1
0	0
1	1
0	0
0	0
0	0
0	0
0	0
0	0
0	0
0	0
0	0
0	0
0	0
1	1
1	1
0	0
0	0
0	0
1	1
1	1
0	0
0	0
0	0
1	1
1	1
1	1
1	1
0	0

1	1
0	0
0	0
1	1
0	0
1	1
0	0
0	0
0	1
1	1
1	1
0	0
0	0
0	1
0	0
1	1
0	0
0	1
1	1
1	1
1	1
1	1
0	0
0	0
1	1
1	1
1	1
1	1
0	0
0	0
1	1
1	1
0	0
1	1
0	0
0	0
0	0
1	1
1	1
1	1

Spoligo 9	Spoligo 10
1	1
0	0
1	1
1	1
0	0
1	1
1	1
0	0
0	0
1	1
1	1
0	0
1	1
0	0
1	1
1	1
1	1
1	1
0	0
0	0
0	0
1	1
1	1
0	0
1	1
1	1
0	0
0	0
0	0
1	1
1	1
1	1
0	0
0	0
0	0
1	0
0	0
0	0
0	0
0	0
0	0
0	0
0	0
0	0
0	0
0	0

0	0
0	0
0	0
0	0
0	0
1	1
0	0
0	0
0	0
0	0
0	0
0	0
0	0
0	0
0	0
0	0
0	0
0	0
1	1
0	0
1	1
0	0
0	0
0	0
0	0
0	0
0	0
0	0
0	0
0	0
0	0
0	0
1	1
1	1
0	0
0	0
0	0
1	1
1	1
0	0
0	0
0	0
1	1
1	1
1	1
1	1
0	0

1	1
0	0
0	0
1	1
0	0
1	1
0	0
0	0
1	1
1	1
1	1
0	0
0	0
1	1
0	0
1	1
0	0
1	1
1	1
1	1
1	1
1	1
0	0
0	0
1	1
1	1
1	1
1	1
0	0
0	0
1	1
1	1
0	0
1	1
0	0
0	0
0	0
1	1
1	1
1	1

Spoligo 11	Spoligo 12
1	1
0	0
1	1
1	1
0	0
1	1
1	1
0	0
0	0
1	0
1	0
0	0
1	0
0	0
1	1
1	1
1	1
1	1
0	0
0	0
0	0
1	1
1	1
0	0
1	1
1	1
0	0
0	0
0	0
1	1
1	1
1	1
0	0
0	0
0	0
1	1
0	0
0	0
0	0
0	0
0	0
0	0
0	0
0	0
0	0
0	0

0	0
0	0
0	0
0	0
0	0
1	1
0	0
0	0
0	0
0	0
0	0
0	0
0	0
0	0
0	0
0	0
0	0
0	0
1	0
0	0
1	1
0	0
0	0
0	0
0	0
0	0
0	0
0	0
0	0
0	0
0	0
0	0
1	1
1	1
0	0
0	0
0	0
1	0
1	1
0	0
0	0
0	0
1	1
1	1
1	1
1	1
0	0

1	1
0	0
0	0
1	1
0	0
1	1
0	0
0	0
1	1
1	1
1	1
0	0
0	0
1	1
0	0
1	1
0	0
1	1
1	1
1	1
1	1
1	1
0	0
0	0
1	1
1	1
1	1
1	1
0	0
0	0
1	1
1	1
0	0
1	1
0	0
0	0
0	0
1	1
1	1
1	1

Spoligo 13	Spoligo 14
0	1
0	0
1	1
0	1
0	0
0	1
1	1
0	0
0	0
1	0
1	0
0	0
1	0
0	0
1	1
0	1
0	1
0	1
0	0
0	0
0	0
0	1
0	1
0	0
1	1
0	1
0	0
0	0
0	0
1	1
1	1
1	1
0	0
0	0
0	0
1	1
0	0
0	0
0	0
0	0
0	0
0	0
0	0
0	0
0	0
0	0

0	0
0	0
0	0
0	0
0	0
0	1
0	0
0	0
0	0
0	0
0	0
0	0
0	0
0	0
0	0
0	0
0	0
0	0
1	0
0	0
0	1
0	0
0	0
0	0
0	0
0	0
0	0
0	0
0	0
0	0
0	0
0	0
0	1
0	1
0	0
0	0
0	0
1	0
0	1
0	0
0	0
0	0
0	1
0	1
0	1
0	1
0	0

0	1
0	0
0	0
1	1
0	0
1	1
0	0
0	0
1	1
0	1
0	1
0	0
0	0
1	1
0	0
0	1
0	0
1	1
0	1
0	1
0	1
1	1
0	0
0	0
0	1
1	1
1	1
0	1
0	0
0	0
1	1
1	1
0	0
1	1
0	0
0	0
0	0
1	1
1	1
0	1

Spoligo 15	Spoligo 16
1	1
0	0
1	1
1	1
0	0
1	1
1	1
0	0
0	0
0	0
0	0
0	0
0	0
0	0
1	1
1	1
1	1
1	1
0	0
0	0
0	0
1	1
1	1
0	0
1	1
1	1
0	0
0	0
0	0
1	1
1	1
1	1
0	0
0	0
0	0
1	1
0	0
0	0
0	0
0	0
0	0
0	0
0	0
0	0
0	0
0	0

0	0
0	0
0	0
0	0
0	0
1	1
0	0
0	0
0	0
0	0
0	0
0	0
0	0
0	0
0	0
0	0
0	0
0	0
0	0
0	0
1	1
0	0
0	0
0	0
0	0
0	0
0	0
0	0
0	0
0	0
0	0
0	0
1	1
1	1
0	0
0	0
0	0
0	0
1	1
0	0
0	0
0	0
1	1
1	1
1	1
1	1
0	0

1	1
0	0
0	0
1	1
0	0
1	1
0	0
0	0
1	1
1	1
1	1
0	0
0	0
1	1
0	0
1	1
0	0
1	1
1	1
1	1
1	1
1	0
0	0
0	0
1	1
1	1
1	1
1	1
0	0
0	0
1	1
1	1
0	0
1	1
0	0
0	0
0	0
1	1
1	0
1	1

Spoligo 17	Spoligo 18
1	1
0	0
1	1
1	1
0	0
1	1
1	1
0	0
0	0
0	0
0	0
0	0
0	0
0	0
1	1
1	1
1	1
1	1
0	0
0	0
0	0
1	1
1	1
0	0
1	1
1	1
0	0
0	0
0	0
1	1
1	1
1	1
0	0
0	0
0	0
1	1
0	0
0	0
0	0
0	0
0	0
0	0
0	0
0	0
0	0
0	0

0	0
0	0
0	0
0	0
0	0
1	1
0	0
0	0
0	0
0	0
0	0
0	0
0	0
0	0
0	0
0	0
0	0
0	0
0	0
0	0
1	1
0	0
0	0
0	0
0	0
0	0
0	0
0	0
0	0
0	0
0	0
0	0
1	1
1	1
0	0
0	0
0	0
0	0
1	1
0	0
0	0
0	0
1	1
1	1
1	1
1	1
0	0

1	1
0	0
0	0
1	0
0	0
1	1
0	0
0	0
1	1
1	1
1	1
0	0
0	0
1	1
0	0
1	1
0	0
1	1
1	1
1	1
1	1
1	1
0	0
0	0
1	1
1	1
1	1
1	1
0	0
0	0
1	1
1	1
0	0
1	1
0	0
0	0
0	0
1	1
1	1
1	1

Spoligo 19	Spoligo 20
1	1
0	0
1	1
1	1
0	0
1	1
1	1
0	0
0	0
0	0
0	0
0	0
0	0
0	0
1	1
1	1
1	1
1	1
0	0
0	0
0	0
1	1
1	1
0	0
1	1
1	1
0	0
0	0
0	0
1	1
1	1
1	1
0	0
0	0
0	0
1	1
0	0
0	0
0	0
0	0
0	0
0	0
0	0
0	0
0	0
0	0

0	0
0	0
0	0
0	0
0	0
1	1
0	0
0	0
0	0
0	0
0	0
0	0
0	0
0	0
0	0
0	0
0	0
0	0
0	0
0	0
1	1
0	0
0	0
0	0
0	0
0	0
0	0
0	0
0	0
0	0
0	0
0	0
1	1
1	1
0	0
0	0
0	0
0	0
1	1
0	0
0	0
0	0
1	1
1	1
1	1
1	1
0	0

1	1
0	0
0	0
1	1
0	0
1	1
0	0
0	0
1	1
1	1
1	1
0	0
0	0
1	1
0	0
1	1
0	0
1	1
1	1
1	1
1	1
1	1
0	0
0	0
1	1
1	1
1	1
1	1
0	0
0	0
1	1
1	1
0	0
1	1
0	0
0	0
0	0
1	1
1	1
1	1

Spoligo 21	Spoligo 22
1	0
0	0
1	1
1	1
0	0
1	1
1	1
0	0
0	0
0	0
0	0
0	0
0	0
0	0
1	1
1	1
1	1
1	1
0	0
0	0
0	0
1	1
1	1
0	0
1	1
1	1
0	0
0	0
0	0
0	0
1	1
1	1
0	0
0	0
0	0
1	1
0	0
0	0
0	0
0	0
0	0
0	0
0	0
0	0
0	0
0	0

0	0
0	0
0	0
0	0
0	0
1	1
0	0
0	0
0	0
0	0
0	0
0	0
0	0
0	0
0	0
0	0
0	0
0	0
0	0
0	0
1	1
0	0
0	0
0	0
0	0
0	0
0	0
0	0
0	0
0	0
0	0
0	0
1	1
1	1
0	0
0	0
0	0
0	0
1	1
0	0
0	0
0	0
1	1
1	1
1	1
1	1
0	0

1	1
0	0
0	0
1	1
0	0
1	1
0	0
0	0
1	1
1	1
1	1
0	0
0	0
1	1
0	0
1	1
0	0
1	1
1	1
1	1
1	1
1	1
0	0
0	0
1	1
1	1
1	1
1	1
0	0
0	0
1	1
1	1
0	0
1	1
0	0
0	0
0	0
1	1
1	1
1	1

Spoligo 23	Spoligo 24
0	1
0	0
1	1
1	1
0	0
1	1
1	1
0	0
0	0
0	0
0	0
0	0
0	0
0	0
1	1
1	1
1	1
1	1
0	0
0	0
0	0
1	1
1	1
0	0
1	1
1	1
0	0
0	0
0	0
0	0
1	1
1	1
0	0
0	0
0	0
1	1
0	0
0	0
0	0
0	0
0	0
0	0
0	0
0	0
0	0
0	0

0	0
0	0
0	0
0	0
0	0
1	1
0	0
0	0
0	0
0	0
0	0
0	0
0	0
0	0
0	0
0	0
0	0
0	0
0	0
0	0
1	1
0	0
0	0
0	0
0	0
0	0
0	0
0	0
0	0
0	0
0	0
0	0
1	1
1	1
0	0
0	0
0	0
0	0
1	1
0	0
0	0
0	0
1	1
1	1
1	1
1	1
0	0

1	1
0	0
0	0
1	1
0	0
1	1
0	0
0	0
1	1
1	1
1	1
0	0
0	0
1	1
0	0
1	1
0	0
1	1
1	1
1	1
1	1
1	1
0	0
0	0
1	1
1	1
1	1
1	1
0	0
0	0
1	1
1	1
0	0
1	1
0	0
0	0
0	0
1	1
1	1
1	1

Spoligo 25	Spoligo 26
1	1
0	0
1	1
1	1
0	0
1	1
1	1
0	0
0	0
1	1
1	1
0	0
1	1
0	0
1	1
1	1
1	1
1	1
0	0
0	0
0	0
1	1
1	1
0	0
1	1
1	1
0	0
0	0
0	0
1	1
1	1
1	1
0	0
0	0
0	0
1	1
0	0
0	0
0	0
0	0
0	0
0	0
0	0
0	0
0	0
0	0

0	0
0	0
0	0
0	0
0	0
1	1
0	0
0	0
0	0
0	0
0	0
0	0
0	0
0	0
0	0
0	0
0	0
0	0
1	1
0	0
1	1
0	0
0	0
0	0
0	0
0	0
0	0
0	0
0	0
0	0
0	0
0	0
1	1
1	1
0	0
0	0
0	0
1	1
1	1
0	0
0	0
0	0
1	1
1	1
1	1
1	1
1	1

1	1
0	0
0	0
1	1
0	0
1	1
0	0
0	0
1	1
1	1
1	1
0	0
1	1
1	1
1	1
1	1
0	0
1	1
1	1
1	1
1	1
1	1
0	0
0	0
1	1
1	1
1	1
1	1
0	0
0	0
1	1
1	1
0	0
1	1
0	0
0	0
0	0
1	1
1	1
1	1

Spoligo 27	Spoligo 28
0	0
0	0
1	1
1	1
0	0
1	1
1	1
0	0
0	0
1	1
1	1
0	0
1	1
0	0
1	1
1	1
1	1
1	1
0	0
0	0
0	0
1	1
1	1
0	0
1	1
1	1
0	0
0	0
0	0
1	1
1	1
1	1
0	0
0	0
0	0
1	1
0	0
0	0
0	0
0	0
0	0
0	0
0	0
0	0
0	0
0	0

0	0
0	0
0	0
0	0
0	0
1	1
0	0
0	0
0	0
0	0
0	0
0	0
0	0
0	0
0	0
0	0
0	0
0	0
1	1
0	0
1	1
0	0
0	0
0	0
0	0
0	0
0	0
0	0
0	0
0	0
0	0
0	0
1	1
1	1
0	0
0	0
0	0
1	1
1	1
0	0
0	0
0	0
1	1
1	1
1	1
1	1
1	1

1	1
0	0
0	0
1	1
0	0
1	1
0	0
0	0
1	1
1	1
1	1
0	0
1	1
1	1
1	1
1	1
0	0
1	1
1	1
1	1
1	1
1	1
0	0
0	0
1	1
1	1
1	1
1	1
0	0
0	0
1	1
1	1
0	0
1	1
0	0
0	0
0	0
1	1
1	1
1	1

Spoligo 29	Spoligo 30
0	0
0	0
1	1
1	1
0	0
1	1
1	1
0	0
0	0
1	1
1	1
0	0
1	1
0	0
1	1
1	1
1	1
1	1
0	0
0	0
0	0
1	1
1	1
0	0
1	1
1	1
0	0
0	0
0	0
1	1
1	1
1	1
0	0
0	0
0	0
1	1
0	0
0	0
0	0
0	0
0	0
0	0
0	0
0	0
0	0
0	0

0	0
0	0
0	0
0	0
0	0
1	1
0	0
0	0
0	0
0	0
0	0
0	0
0	0
0	0
0	0
0	0
0	0
0	0
1	1
0	0
1	1
0	0
0	0
0	0
0	0
0	0
0	0
0	0
0	0
0	0
0	0
0	0
1	1
1	1
0	0
0	0
0	0
1	1
1	1
0	0
0	0
0	0
1	1
1	1
1	1
1	1
1	1

1	1
0	0
0	0
1	1
0	0
1	1
0	0
0	0
1	0
1	1
1	1
0	0
1	1
1	0
1	1
1	1
0	0
1	0
1	1
1	1
1	1
1	1
0	0
0	0
1	1
1	1
1	1
1	1
0	0
0	0
1	1
1	1
0	0
1	1
0	0
0	0
0	0
1	1
1	1
1	1

Spoligo 31	Spoligo 32
1	1
0	0
1	1
1	1
0	0
1	1
1	1
0	0
0	0
1	1
1	1
0	0
1	1
0	0
1	1
1	1
1	1
1	1
0	0
0	0
0	0
1	1
1	1
0	0
1	1
1	1
0	0
0	0
0	0
1	1
1	1
1	1
0	0
0	0
0	0
1	1
0	0
0	0
0	0
0	0
0	0
0	0
0	0
0	0
0	0
0	0

0	0
0	0
0	0
0	0
0	0
1	1
0	0
0	0
0	0
0	0
0	0
0	0
0	0
0	0
0	0
0	0
0	0
0	0
1	1
0	0
1	1
0	0
0	0
0	0
0	0
0	0
0	0
0	0
0	0
0	0
0	0
0	0
1	1
1	1
0	0
0	0
0	0
1	1
1	1
0	0
0	0
0	0
1	1
1	1
1	1
1	1
1	1

1	1
0	0
0	0
1	1
0	0
1	1
0	0
0	0
0	0
1	1
1	1
0	0
1	1
0	0
1	1
1	1
0	0
0	0
1	1
1	1
1	1
1	1
0	0
0	0
1	1
1	1
1	1
1	1
0	0
0	0
1	1
1	1
0	0
1	1
0	0
0	0
0	0
1	1
1	1
1	1

Spoligo 33	Spoligo 34
0	0
0	0
0	0
0	0
0	0
0	0
0	0
0	0
0	0
0	0
0	0
0	0
0	0
0	0
0	0
0	0
0	0
0	0
0	0
0	0
0	0
0	0
0	0
0	0
0	0
0	0
0	0
0	0
0	0
0	0
0	0
0	0
0	0
0	0
0	0
0	0
0	0
0	0
0	0
0	0
0	0
0	0
0	0
0	0
0	0
0	0

0	0
0	0
0	0
0	0
0	0
0	0
0	0
0	0
0	0
0	0
0	0
0	0
0	0
0	0
0	0
0	0
0	0
0	0
0	0
0	0
0	0
0	0
0	0
0	0
0	0
0	0
0	0
0	0
0	0
0	0
0	0
0	0
0	0
0	0
0	0
0	0
0	0
0	0
0	0
0	0
0	0
0	0
0	0
0	0
0	0
0	0
0	0

0	0
0	0
0	0
0	0
0	0
0	0
0	0
0	0
0	0
0	0
0	0
0	0
0	0
0	0
0	0
0	0
0	0
0	0
0	0
0	0
0	0
0	0
0	0
0	0
0	0
0	0
0	0
0	0
0	0
0	0
0	0
0	0
0	0
0	0
0	0
0	0
0	0
0	0
0	0
0	0

Spoligo 35	Spoligo 36
0	0
1	1
0	0
0	0
1	1
1	1
0	0
1	1
1	1
0	0
0	0
1	1
0	0
1	1
0	0
0	0
0	0
0	0
1	1
1	1
1	1
0	0
0	0
1	1
0	0
0	0
1	1
1	1
1	1
0	0
0	0
0	0
1	1
1	1
1	1
0	0
1	1
1	1
1	1
1	1
1	1
1	1
1	1
1	1
1	1
1	1

1	1
1	1
1	1
1	1
1	1
0	0
1	1
1	1
1	1
1	1
1	1
1	1
1	1
1	1
1	1
1	1
1	1
1	1
0	0
1	1
0	0
1	1
1	1
1	1
1	1
1	1
1	1
1	1
1	1
1	1
1	1
1	1
0	0
0	0
1	1
1	1
1	1
0	0
0	0
1	1
1	1
1	1
0	0
0	0
0	0
0	0
0	0

0	0
1	1
1	1
0	0
1	1
0	0
0	0
1	1
0	0
0	0
0	0
1	1
0	0
0	0
0	0
0	0
1	1
0	0
0	0
0	0
0	0
0	0
1	1
1	1
0	0
0	0
0	0
0	0
1	1
1	1
0	0
0	0
1	1
0	0
1	1
1	1
1	1
0	0
0	0
0	0

Spoligo 37	Spoligo 38
1	1
1	1
1	1
1	1
1	1
1	1
1	1
1	1
1	1
1	1
1	1
1	1
1	1
1	1
1	1
1	1
1	1
1	1
1	1
0	1
1	1
1	1
1	1
1	1
1	1
1	1
1	1
1	1
1	1
1	1
1	1
1	1
1	1
1	1
1	1
1	1
1	1
1	1
1	1
1	1
1	1
1	1
1	1
1	1
1	1
1	1

1	1
1	1
1	1
1	1
1	1
1	1
1	1
1	1
1	1
1	1
1	1
1	1
1	1
1	1
1	1
1	1
1	1
1	1
1	1
1	1
1	1
1	1
1	1
1	1
1	1
1	1
1	1
1	1
1	1
1	1
1	1
1	1
1	1
1	1
1	1
1	1
1	1
1	1
1	1
1	1
1	1
1	1
1	1
1	1
1	1
1	1
1	1

1	1
1	1
1	1
1	1
1	1
1	1
1	1
1	1
0	0
1	1
1	1
1	1
1	1
0	0
1	1
1	1
1	1
0	0
1	1
1	1
1	1
1	0
1	1
1	1
1	1
1	1
1	1
1	1
1	1
1	1
1	1
1	1
1	1
1	1
1	1
1	1
1	1
1	1
1	1
1	1

Spoligo 39	Spoligo 40
1	1
1	1
1	1
1	1
1	1
1	1
1	1
1	1
1	1
1	1
1	1
1	1
1	1
1	1
1	1
1	1
1	0
1	1
1	1
1	1
1	1
1	1
1	1
1	1
1	1
1	1
1	1
1	1
1	1
1	1
1	1
1	1
1	1
1	1
1	1
1	1
1	1
1	1
1	1
1	1
1	1
1	1
1	1
1	1
1	1
1	1

1	1
1	1
1	1
1	1
1	1
1	1
1	1
1	1
1	1
1	0
1	1
1	1
1	1
1	1
1	0
1	1
1	1
1	1
1	1
1	1
1	1
1	1
1	1
1	1
1	1
1	1
1	1
1	1
1	1
1	1
1	1
1	1
1	0
1	1
1	1
1	1
1	1
1	1
1	0
1	1
1	1
1	1
1	1
1	1
1	1
1	1
1	1

1	1
1	1
1	1
0	0
1	1
1	1
1	1
1	1
0	1
1	1
1	1
1	1
1	1
0	1
1	1
1	1
1	1
0	1
1	1
1	1
1	1
1	1
1	1
1	1
1	1
1	1
1	1
1	1
1	1
1	1
1	1
1	1
1	1
1	1
1	1
1	1
1	1
1	1
1	1
1	1

Spoligo 41	Spoligo 42
1	1
1	1
1	1
1	1
1	1
1	1
1	1
1	1
1	1
1	1
1	1
1	1
1	1
1	1
1	1
1	1
1	1
1	1
1	1
1	1
1	1
1	1
1	1
1	1
1	1
1	1
1	1
1	1
1	1
1	1
1	1
1	1
1	1
1	1
1	1
1	1
1	1
1	1
1	1
1	1
1	1
1	1
1	1
1	1
1	1
1	1

1	1
1	1
1	1
1	1
1	1
1	1
1	1
1	1
1	1
1	1
1	1
1	1
1	1
1	1
1	1
1	1
1	1
1	1
1	1
1	1
1	1
1	1
1	1
1	1
1	1
1	1
1	0
1	1
1	1
1	1
1	1
1	0
1	1
1	1
1	1
1	1
1	1
1	1
1	1
1	1
1	1
1	1
1	1
1	1
1	1
1	1
1	1

1	1
1	1
1	1
0	0
1	1
1	1
1	1
1	1
1	1
1	1
1	1
1	1
1	1
1	1
1	1
1	1
1	1
1	1
1	1
1	1
1	1
1	1
1	1
1	1
1	1
1	1
1	1
1	1
1	1
1	1
1	1
1	1
1	1
1	1
1	1
1	1
1	1
1	1
1	1
1	1

Spoligo 43	QUB-11b
1	4
1	0
1	4
1	2
1	6
1	3
1	1
1	5
1	6
1	0
1	0
1	7
1	0
1	5
1	3
1	3
1	3
1	4
1	4
1	6
1	4
1	2
1	3
1	4
1	7
1	2
1	7
1	5
1	5
1	2
1	4
1	4
1	6
1	4
1	7
1	3
1	6
1	5
1	6
1	5
1	10
1	6
1	6
1	6
1	6
1	6

1	6
1	6
1	6
1	6
1	6
1	2
1	6
1	6
1	6
1	7
1	4
1	6
1	6
1	6
1	7
1	7
1	6
1	6
1	0
1	5
1	2
1	6
1	6
1	5
1	6
1	6
1	5
1	6
1	5
1	5
1	6
1	5
1	2
1	2
1	5
1	6
1	6
1	3
1	2
1	5
1	6
1	7
1	3
1	3
1	3
1	3
1	0

1	3
1	6
1	6
1	3
1	5
1	4
1	7
1	6
1	4
1	3
1	3
1	6
1	0
1	4
1	0
1	3
1	6
1	4
1	3
1	3
1	3
1	1
1	6
1	7
1	2
1	1
1	2
1	6
1	7
1	4
1	4
1	4
1	3
1	3
1	7
1	5
1	6
1	3
1	3
1	3

QUB-18	QUB-26
5	7
10	9
5	6
5	11
8	8
5	11
8	9
8	8
7	8
0	4
0	4
10	7
0	4
8	7
5	8
7	11
0	4
0	6
10	3
10	8
10	7
5	5
5	10
8	7
5	8
5	9
10	8
7	8
8	8
2	6
3	5
5	4
8	8
9	8
10	9
5	4
6	7
8	8
10	9
10	8
10	2
3	8
3	8
3	8
3	8
8	8

3	8
3	8
3	8
3	8
3	8
5	12
3	8
3	8
3	1
6	7
8	8
3	8
3	8
3	8
6	7
10	2
3	8
3	8
0	4
8	8
6	12
3	8
3	8
8	8
10	8
3	1
6	8
10	8
10	7
8	8
3	8
6	8
5	11
6	12
8	8
3	8
3	8
0	4
5	11
8	8
3	8
10	7
4	12
4	12
4	12
4	12
0	2

4	12
10	7
10	7
9	8
12	8
6	5
11	8
11	8
5	9
4	12
4	12
8	8
0	2
5	9
0	2
4	12
10	7
5	9
4	12
4	2
6	5
9	6
12	2
12	7
1	1
9	8
4	8
5	11
9	8
11	7
5	8
5	3
12	9
2	3
9	8
12	9
12	8
5	9
10	6
5	0

QUB-4156	QUB-1895
1	4
4	4
2	4
2	4
4	4
2	4
3	4
2	4
2	4
2	2
2	2
4	2
2	2
3	4
2	4
2	4
2	4
2	4
4	4
2	4
4	2
2	4
2	4
2	4
2	4
2	4
2	4
2	4
2	4
2	4
2	4
2	2
2	2
3	4
4	4
2	4
4	4
2	4
4	4
3	2
4	2
2	4
2	4
2	4
2	4
2	4

2	4
2	4
2	4
2	4
2	4
2	4
2	4
2	4
2	4
4	4
2	4
2	4
2	4
2	4
4	4
4	1
2	4
2	4
2	4
2	4
2	4
2	4
2	4
2	4
4	4
2	4
2	4
4	4
4	2
2	4
2	4
2	4
2	4
2	4
2	4
2	4
2	4
2	4
2	4
2	4
2	4
4	2
2	4
2	4
2	4
2	4
2	4

2	4
1	2
1	2
3	2
2	4
2	3
4	0
4	4
2	3
2	4
2	4
2	4
2	4
2	3
2	4
2	4
1	2
2	3
2	4
2	4
2	4
3	4
4	2
4	2
2	4
3	2
2	4
2	4
2	4
4	2
2	4
2	4
4	4
2	4
2	4
3	4
6	4
2	3
3	4
2	4

MIRU26	MIRU31
4	3
8	5
5	3
7	4
8	5
7	3
1	3
8	5
8	5
5	3
5	3
8	5
5	3
8	5
3	3
6	3
3	3
5	3
7	3
7	5
7	4
7	3
8	3
7	5
5	3
6	4
5	5
6	5
7	5
5	3
5	3
5	5
7	5
5	6
5	4
5	3
5	5
7	5
5	5
7	5
9	5
8	5
8	5
8	5
8	5
8	5

8	5
8	5
8	5
8	5
8	5
4	3
8	5
8	5
8	5
7	5
7	5
8	5
8	5
8	5
7	5
8	5
8	5
8	5
6	3
8	5
7	2
8	5
8	5
8	5
8	5
7	5
8	5
8	5
8	5
8	5
8	5
8	5
7	3
7	2
6	5
8	5
8	5
5	3
6	3
8	5
8	5
8	5
7	3
7	3
7	3
7	3
6	3

7	3
6	5
6	5
6	3
4	5
6	2
7	8
8	5
8	2
4	3
7	3
7	5
6	3
4	2
7	3
8	3
7	5
4	2
8	3
8	3
8	4
2	3
8	5
10	5
4	3
2	3
7	4
10	3
8	4
10	5
6	3
5	3
10	5
6	3
10	7
7	5
7	5
3	3
1	3
7	3

MIRU10	MIRU40
3	3
3	3
2	4
2	3
3	3
2	3
5	3
3	4
3	3
2	2
2	2
3	3
2	2
3	1
2	2
2	2
2	2
2	4
3	3
3	3
3	3
2	2
2	3
2	3
2	3
2	4
3	3
3	3
3	3
4	1
2	3
2	3
3	3
3	3
3	3
3	2
3	3
3	3
1	3
3	3
3	3
3	3
3	3
3	3
3	3
2	3

3	3
3	3
3	3
3	3
3	3
2	3
3	3
3	3
3	3
1	4
3	3
3	3
3	3
3	3
1	4
3	3
3	3
3	3
2	2
3	4
2	3
3	3
3	3
3	4
2	3
3	3
3	3
2	3
3	3
3	4
3	3
3	3
2	2
2	3
3	4
3	3
3	3
2	2
2	2
3	4
3	3
3	3
2	3
2	3
2	3
2	3
2	4

2	3
3	3
3	3
4	4
3	3
2	3
3	3
3	1
2	3
2	3
2	3
3	3
2	2
2	3
2	2
2	3
3	3
2	3
2	3
2	3
2	3
5	2
3	3
3	3
2	3
5	2
2	3
2	3
3	4
3	3
2	3
2	3
3	3
2	3
3	3
3	3
3	5
2	3
5	2
2	3

Mtub21	Mtub04
3	2
5	4
3	2
3	2
4	2
2	2
3	3
5	4
5	4
1	4
1	4
4	4
1	4
4	3
3	2
2	3
1	2
1	3
3	2
5	4
4	4
3	2
3	1
5	4
3	2
3	2
2	4
5	4
5	3
3	5
3	2
3	2
5	5
4	3
4	3
2	3
2	4
5	4
4	4
4	4
4	4
8	4
8	4
8	4
8	4
5	5

8	4
8	4
8	4
8	4
8	4
3	2
8	4
8	4
8	4
4	4
5	3
8	4
8	4
8	4
4	4
4	4
8	4
8	4
1	4
7	4
3	2
8	4
8	4
7	4
4	2
8	4
5	4
4	2
4	4
7	4
8	4
5	4
3	2
3	2
7	4
8	4
8	4
1	4
3	2
7	4
8	4
4	4
2	1
2	1
2	1
2	1
1	4

2	1
1	4
1	4
3	2
5	4
3	2
4	4
4	4
3	2
2	1
2	1
5	4
1	4
3	2
1	4
2	1
1	4
3	2
2	1
2	1
3	3
3	3
4	4
4	4
3	2
2	3
3	2
3	1
5	4
4	4
3	2
2	2
5	4
3	2
5	4
4	3
4	3
3	2
3	3
2	2

ETR-F
0
2
1
0
2
1
0
2
0
1
1
2
1
1
1
1
2
2
2
2
2
1
1
2
1
0
2
2
2
1
0
1
2
1
3
2
2
2
3
1
2
2
2
2
3
3

2
2
2
2
2
1
2
2
2
1
2
2
1
2
2
2
2
1
1
2
2
2
0
1
2
2
2
2
2
2
2
2
2
2
2
1
2
2
2
3
2
2
1
1
1
1
3

1
2
2
2
2
1
2
2
1
1
1
1
3
1
3
1
2
1
1
1
1
1
2
0
1
1
1
1
2
2
1
1
2
0
1
3
2
1
1
0
